# Family Outcomes of a Community-Based Trial of Project ImPACT

**DOI:** 10.3390/bs16010064

**Published:** 2025-12-31

**Authors:** Sarah R. Rieth, Marissa Chemotti, Carmen Orendain Soto, Sarah F. Vejnoska, Scott Roesch, Amber Fitzgerald, Sarah Dufek, Aubyn C. Stahmer

**Affiliations:** 1 Department of Child and Family Development, College of Education, San Diego State University, San Diego, CA 92182, USA; 2 Child and Adolescent Services Research Center, San Diego, CA 92122, USA; mchemotti@sdsu.edu (M.C.);; 3 Department of Psychiatry and Behavioral Sciences, MIND Institute, School of Medicine, University of California, Davis, Sacramento, CA 95819, USA; sfvejnoska@health.ucdavis.edu (S.F.V.); afitzgerald@health.ucdavis.edu (A.F.); sadufek@health.ucdavis.edu (S.D.); astahmer@health.ucdavis.edu (A.C.S.); 4 Department of Psychology, College of Science, San Diego State University, San Diego, CA 92182, USA

**Keywords:** autism, early intervention, naturalistic developmental behavioral intervention, parent coaching, Project ImPACT, community-based trial

## Abstract

Caregiver-mediated approaches in early intervention can provide impactful support for families of young children with social communication needs. Project ImPACT (PI), a caregiver-mediated naturalistic developmental behavioral intervention, was tested for effectiveness in public early intervention (EI) programs in a randomized waitlist-control community trial across California. Participants included EI service providers (n = 47) and caregiver–child dyads (n = 125; ages 14–32 months). Families received services-as-usual (SAU) or PI following provider training in PI. Multilevel models were used to examine provider coaching, caregiver–child interactions, caregiver PI strategy use, parenting stress, self-efficacy in parenting, and child social communication outcomes across approximately four months of services. Provider use of evidence-based coaching significantly improved after PI training. Caregivers who received PI showed greater gains in some domains of parent–child interaction; PI fidelity scores, stress, and self-efficacy did not differ by condition. Child communication outcomes improved over time in both groups, but differences between conditions were not detected during the study time period. Training community EI providers in PI improved coaching quality and enhanced caregiver–child interaction, demonstrating feasible, scalable use of PI in community settings. Differential child-level effects were not detected, underscoring the need for larger samples and longitudinal follow-up.

## 1. Introduction

Developmental differences early in childhood, especially social communication challenges, often indicate a high likelihood of future diagnoses of conditions such as autism (Bradshaw et al., 2021; Estes et al., 2015; Lazenby et al., 2016; Miller & Ozonoff, 2020). In the United States, one in 6 children between 3 and 17 years old has one or more developmental disabilities, representing a significant public health need (Cogswell et al., 2022; Zablotsky et al., 2019). Social communication delays and behavioral challenges in infancy are associated with ongoing linguistic, educational, and social difficulties If unsupported in early development, these toddlers are likely to have support needs when they reach school age (Delehanty et al., 2018; Martelli et al., 2025).

Often, children with early regulation differences, communication delays, difficulty engaging in social relationships, loss of previously acquired skills, and socio-behavioral difficulties later receive a diagnosis of autism. Autism diagnoses have increased at dramatic rates in the last decade, with a current incidence of 1 in 36 children (Baio et al., 2018; Maenner et al., 2023). Greater awareness of autism and improvement in diagnostic tools have led to stable identification of toddlers as early as 14 months of age (Pierce et al., 2019). As such, communities are striving to implement effective programs for toddlers presenting with concerns that may indicate autism, especially programs that are matched to the child’s developmental level and the family’s unique culture and support needs. Although evidence-based practices for autism exist (National Autism Center, 2015; Steinbrenner et al., 2020), practices specifically designed for toddlers have generally not been effectively translated into community settings where the majority of children can benefit (Aranbarri et al., 2021). On local and national levels, there are calls for capacity building for this population of young children experiencing social communication challenges.

### 1.1. The Need for Caregiver-Mediated Interventions in Community Settings

In addition to supporting children as early in development as possible, it has long been established that caregiver involvement in early intervention (EI) is crucial for optimal child outcomes (Institute of Medicine and National Research Council, 2000; Maglione et al., 2012). In caregiver-mediated interventions, caregivers learn to use intervention strategies and are taught to integrate evidence-based strategies into their daily routines with their young children. In addition to effectively promoting child progress, caregiver-mediated intervention may decrease stress in some parents (Estes et al., 2014). While efficacy data for early caregiver-mediated autism interventions are promising (Conrad et al., 2021; Pan et al., 2023; Reichow et al., 2024), there remains a need for interventions with demonstrated effectiveness in community settings where the majority of families receive services. There continues to be limited capacity to effectively serve families of toddlers at high likelihood of autism in the community, and available services often lack consistent quality. Therefore, it is critical to test the effectiveness of sustainable methods to deliver quality, early caregiver-mediated intervention for toddlers presenting with behaviors related to autism.

Multiple challenges to the delivery of evidence-based caregiver-mediated intervention exist in community-based services. Active parent participation is not widely implemented in these settings, despite being a value and mandate of publicly implemented EI systems. Data indicate that a majority of publicly funded EI sessions involve the caregiver playing a passive rather than active role (Aranbarri et al., 2021; Campbell & Sawyer, 2007; Coogle et al., 2013). Additionally, current training for EI providers equips them to work directly with children with general developmental delays but not to build caregiver capacity, coach caregivers effectively (Dunst & Bruder, 2015) or utilize autism-specific strategies to support development (Ingersoll et al., 2012). The field of EI has attempted some focus on how to optimally coach caregivers (Dunst & Bruder, 2015; Fettig & Barton, 2014; Friedman et al., 2012) as well as how to address characteristics related to autism in the context of EI (Aranbarri et al., 2021; Stahmer et al., 2017), but considerable work is needed in these areas to effectively move these values to day-to-day community practice (Pellecchia et al., 2023).

### 1.2. Using a Community-Participatory Approach to Improve PMI in Community EI

The BRIDGE Collaborative is a community–academic partnership, composed of clinicians, parents, researchers, and funding agency representatives that share a common goal of ensuring families receive high-quality, evidence-based EI services in the community (Brookman-Frazee et al., 2012b). BRIDGE is an acronym that represents the shared early development values of the group: Bond, Regulate, Interact, Develop, Guide, and Engage. Early on, the Collaborative received federal funding to adapt an evidence-based, parent coaching intervention to fit with the characteristics and best practices of publicly funded EI programs (Rieth et al., 2018). The resulting intervention, Project ImPACT for Toddlers, represented a collaborative adaptation of Project ImPACT (Improving Parents as Communication Teachers; PI; Ingersoll & Dvortscak, 2019). Project ImPACT is an evidence-based, Naturalistic Developmental Behavioral Intervention (NDBI; Schreibman et al., 2015) that focuses on equipping caregivers to support their child’s social and communication development. This intervention was chosen based on its fit with state-funded services and the principles of quality EI (for example, as advocated by Schertz et al., 2011). PI for Toddlers was created to meet the needs of providers and agencies serving the youngest children with social communication challenges and their families. Specific adaptations were made to address EI principles and practice recommendations, match community values, ensure fit with the Spanish-speaking community, and facilitate provider training in active caregiver coaching strategies.

A quasi-experimental pilot test of PI for Toddlers demonstrated positive results compared to services-as-usual when PI for Toddlers was delivered by community-based service providers (Stahmer et al., 2020). Specifically, parents receiving PI for Toddlers demonstrated greater gains in their ability to engage in supportive parent–child interactions than parents receiving typical services across the 3-month intervention period. Children receiving PI for Toddlers demonstrated gains in standardized social and communication measures with an average effect size of 0.55 (SD = 0.38) across the intervention period and an effect size of 0.87 (SD = 0.51) from the start of intervention to follow-up measurement (3 months after the end of intervention). Children in services-as-usual made gains with an average effect size of 0.18 (SD = 0.19) during intervention and 0.29 (SD = 0.26) at the follow-up point. Implementation measures demonstrated high fidelity to PI for Toddlers coaching strategies by providers, high feasibility for community implementation, and strong satisfaction with the manual and training methods. Based on the promising results of this pilot study, a larger effectiveness trial of PI for Toddlers in community settings was warranted.

Adaptations from the first-edition manual of PI, from which PI for Toddlers was adapted, were later integrated into the second edition of the PI manual (Ingersoll & Dvortscak, 2019). As such, the BRIDGE Collaborative opted to move forward with the second edition of the PI manual for community use with toddlers, and we now refer to the intervention as “PI”.

### 1.3. Community Trial

The current community trial tests the effectiveness of PI within community-based EI programs throughout California. Service providers employed by community EI agencies serving young children with or at high likelihood of autism received training in how to provide PI to families at different time periods. The study is registered in the Society for Research on Educational Effectiveness’s Registry of Efficacy and Effectiveness Studies (#10400). A randomized waitlist-control design was used to examine the influence of PI at the service provider, parent, and child levels. Specific questions include:

(1) Provider: To what degree do EI service providers deliver effective caregiver coaching after receiving training in PI?
(2) Parent: To what degree do caregivers demonstrate change after receiving PI in community services in:
  - applying PI techniques with their child;
  - engaging in developmentally supportive interactions with their child;
  - reported stress;
  - reported feelings of parenting self-efficacy.
(3) To what degree does PI delivered in community services affect children’s social and communication skills?

## 2. Materials and Methods

A randomized waitlist-control trial was conducted with community EI providers paired with families of children 12–36 months old with needs related to social communication development. Participating service providers were employed by agencies providing publicly funded EI services in the community. At the beginning of participation, providers were randomly assigned to a waitlist group of varying lengths. That is, they were randomized to provide services as they typically would and submit data for two or three families (4 months per family). Providers were assigned to a condition using a restricted randomization procedure to maintain balance across groups during rolling enrollment. Specifically, the first participant in each consecutive pair was randomly assigned to either the two or three usual care families (via coin flip), and the subsequent participant was automatically assigned to the opposite condition. This approach aimed to create equal group sizes while retaining an element of randomization in time in the control condition and also being feasible for rolling community recruitment and timelines. After completing the services-as-usual phase with their assigned number of families (two or three), service providers then received PI training. Following training, service providers delivered PI to three or two families (i.e., providers who completed services with two services-as-usual families were assigned to complete PI with three families; providers who completed services-as-usual with three families completed PI with two families). A comparison between assigned groups indicated significantly different amounts of time in the services-as-usual control (baseline) condition based on group assignment (M = 164 days for the 2-family services-as-usual group, M = 277 days for the 3-family services-as-usual group, *p* < 0.05).

Participating families were referred to provider agencies due to eligibility for EI services as established by the local Part C funding agency or available insurance. Outcomes are compared across children and families who received services-as-usual versus PI from the same local EI service providers.

### 2.1. Sample

A total of 47 service providers from EI agencies and 125 caregiver/child dyads participated in the community trial. Service providers were recruited through local EI agencies, and caregiver/child dyads were recruited through providers participating in the study. Families participated in either the services-as-usual or PI condition, based on whether or not the provider had received PI training at the time of the family’s participation. Random assignment of caregiver/child dyads to providers was not possible or appropriate because the intervention was being delivered in the context of the existing public service system. Informed consent was obtained from all subjects involved in the study.

Study recruitment began in March 2020. The onset of the COVID-19 pandemic in the early stages of the study resulted in a brief pause in research activity. The study resumed with a fully virtual protocol in August 2020, at which time recruitment began anew, and all previously recruited participants were considered discontinued. All data reported here were collected beginning in August 2020. Study recruitment and activities were heavily impacted by the disruptions to the EI service system caused by COVID-19.

### 2.2. Study Eligibility and Recruitment

Initially, the study criteria included participants exclusively in the San Diego and Sacramento regions based on the locations of the research team. With the transition to remote study participation during the COVID-19 pandemic, participation expanded to EI services across the state of California. However, the majority of participants were drawn from the two primary catchment areas of San Diego and Sacramento.

#### 2.2.1. Service Providers

Eligible providers worked at agencies that provided EI services in California, whose agency leader verbally agreed to support participation. Providers were required to have a bachelor’s degree equivalent or higher, at least one year of experience at their current agency, plans to continue working at the current agency for at least one more year, a toddler (ages 12–36 months) caseload of at least two toddlers with social communication goals, at least one year of experience working with children with or at high likelihood of autism, and no previous use of PI or similar manualized NDBI programs with families.

Providers were recruited with the assistance of state funders (local Regional Centers who administer the California Part C program), County Departments of Education, and relationships with local stakeholder groups (e.g., BRIDGE Collaborative, Project ECHO at University of California, Davis). Interested providers completed a brief online eligibility survey and then were contacted by the study team.

#### 2.2.2. Families

Eligible caregiver–child dyads included children who were 12–32 months old at the time of enrollment, who qualified for EI services from their local Regional Center or via insurance, who had goals or concerns related to social communication development, and whose primary caregiver spoke English or Spanish.

Children/families were excluded from the study if (1) the child did not have goals related to social communication development with the participating provider, (2) the child had known significant organic brain damage or major medical problems, or (3) the family had prior experience receiving or had plans to begin receiving PI services with another agency in the community or a similar manualized NDBI program.

Providers and/or agency staff introduced the study opportunity to eligible families on caseloads or families newly referred to the agency. For families willing to be contacted, members of the research team reached out via phone, text, or email to answer questions and complete study consent forms.

### 2.3. Participation Procedures

#### 2.3.1. Service Provider Participation Procedures

**Figure 1:**
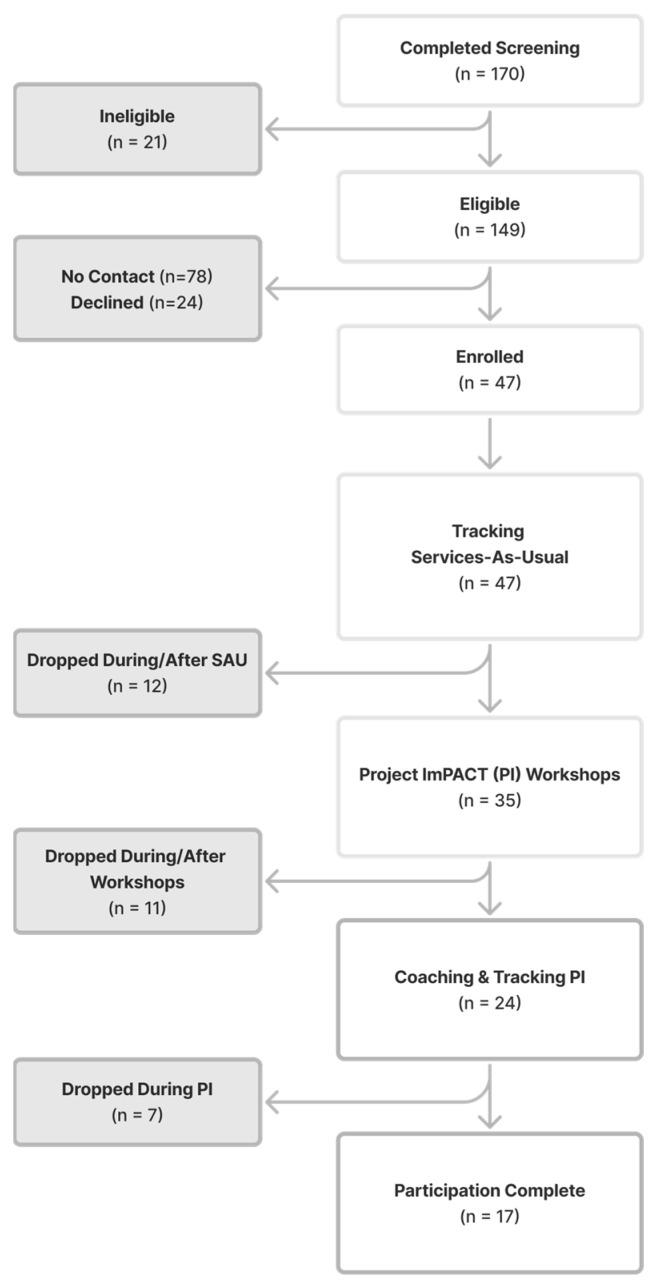
Service Provider Consort Diagram.

Upon enrollment, providers completed a demographics questionnaire to provide information on their background and training experiences. Specific measures collected from providers during services-as-usual and PI service delivery are detailed below. Once providers completed services with their assigned number of services-as-usual families, they participated in PI training with the study team. Full details of PI training activities are included below. Providers then enrolled children referred/receiving services after their PI training into the PI condition of the trial. Figure 1 illustrates the participation process for service providers, including attrition across the study timeline.

#### 2.3.2. Family Participation Procedures

Upon enrollment, families completed intake assessments with the study team and completed questionnaires. Services were followed across the approximately four months of receiving either services-as-usual or PI through video recordings. After four months, families completed exit assessments. The four-month timeline was selected based on the designed length of the PI service (generally 12 weeks).

**Figure 2:**
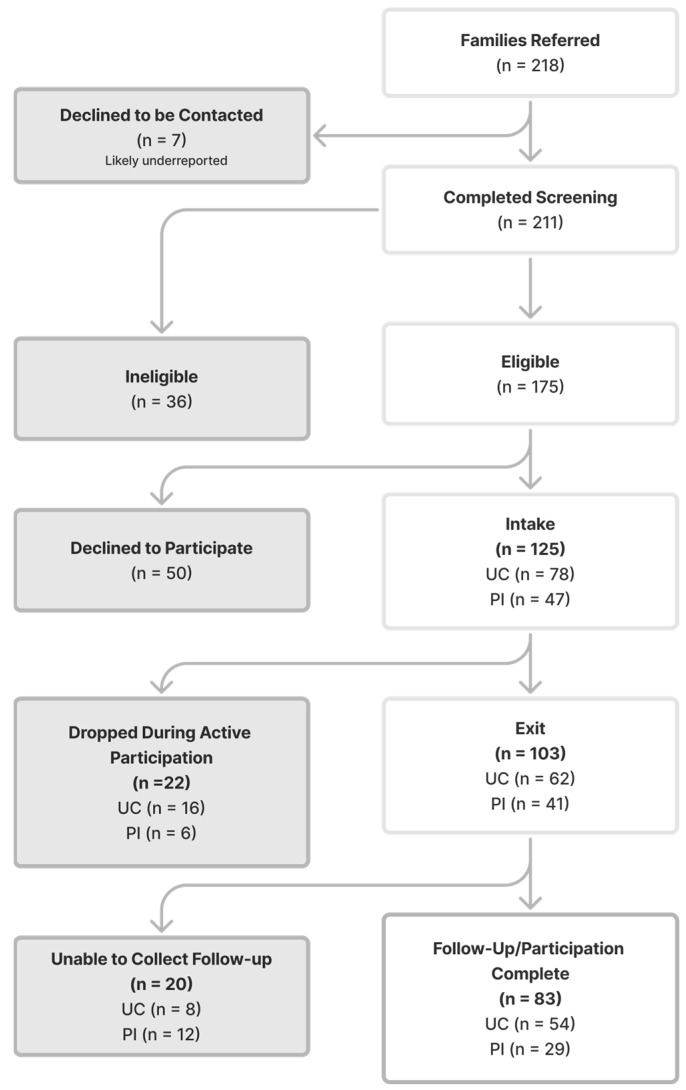
Family Consort Diagram.

At the child’s 3rd birthday, families were sent a follow-up questionnaire to assess the child’s continued developmental progress, including the DP-3 Communication scale. If the child’s 3rd birthday was within one month of the exit date, separate follow-up measures were not collected. Figure 2 illustrates the participation process for families, including attrition across the study timeline.

### 2.4. PI Training

Service provider training consisted of an initial 20 h of instruction completed across a 6-week period, followed by individual coaching on PI implementation to fidelity by senior research team members in coordination with the ImPACT program developers.

#### 2.4.1. Initial Training

The initial 20 h of training consisted of an online tutorial of PI and six virtual synchronous small-group workshops that each lasted 2.5 h. Providers independently viewed approximately six hours of interactive, didactic content online in small, topical chunks. These tutorials were created by the PI developers (Beginner Course, available at www.project-impact.org). Each week, providers viewed the online tutorial for 1–4 topics/strategies (assignments ranging from 30 to 90 min per week) and then attended a 2.5 h virtual group workshop regarding those topics. The virtual group workshops consisted of 3–4 providers and 2 trainers in each session. In workshops 2 through 5, a volunteer family joined the training, and providers practiced coaching the parent on the week’s topic while receiving feedback from the trainers and other trainees. Other group workshop activities included a brief review of the online modules, as well as activities and role-plays to practice working collaboratively with parents.

#### 2.4.2. Individual Coaching

After providers completed the six weeks of initial training, they began using PI with families whom they saw in the context of their routine services. During this phase, PI trainers provided individual coaching on providers’ implementation of PI with families through video review. Trainers met with providers briefly after each video to discuss feedback and fidelity of implementation. Individual coaching sessions continued with providers until they met fidelity criteria (80% or better on the Fidelity Checklist) across three different topics/areas of PI (one at each level of the PI pyramid).

#### 2.4.3. Training Team

PI training was provided to participants by experienced clinicians who were part of the research team. Clinical trainers were EI professionals who hold MA degrees or higher and have at least 5 years of experience working with families of young children with autism. All trainers completed the PI online tutorial, attended an ‘Advanced Workshop’ two-day class offered by the PI developers, and met fidelity criteria while delivering PI to at least two families while receiving clinical supervision from the senior research team in coordination with the program developers. Throughout the study, trainers met bi-weekly to check fidelity and feedback alignment, discuss providers in training and make any necessary adjustments to the training protocol or activities.

### 2.5. Measures

A multi-method assessment battery was used to characterize participants at all levels and examine change over time.

#### 2.5.1. Service Provider Measures

##### Provider Characterization Measures

Demographics. At enrollment, providers complete a demographics survey including their experience in their field (e.g., years of experience), education, training in specific methodologies, and theoretical background (e.g., behavioral and developmental training). This survey was developed by the authors for the purposes of this study.

Satisfaction and Implementation Survey. After completion of PI with at least two families or upon exit from the study, service providers were asked to complete a Satisfaction and Implementation Survey regarding their experience receiving PI training and implementing the model. The 31-item survey asked providers to rate a series of statements from 1 to 5 related to their satisfaction with PI training (10 items), their beliefs around the impact of the PI model (7 items), and the implementation of the approach (12 items). These items were rated on a 1–5 scale, where 1 indicated very dissatisfied/not at all/completely disagree and 5 indicated very satisfied/very much/completely agree. The means of each subsection (training, impact, and implementation) are reported. Additionally, three questions asked providers the most useful components of the PI model, the most difficult components to teach, and whether they would recommend the training and/or PI to other service providers.

##### Provider-Dependent Variables

Coaching strategies. The Parent Empowerment and Coaching in Early Intervention (PEACE; Pellecchia et al., 2020) behavioral coding framework was used to rate provider use of evidence-based coaching strategies during sessions. PEACE was coded from the bi-monthly video recordings of sessions facilitated by the research team. ICCs for PEACE items ranged from 0.57 to 0.98, with an average of 0.86, indicating moderate to good reliability for coding (Cicchetti, 1994). The average of all items in the PEACE coding framework from the providers’ top three videos was used for analyses as an overall indicator of coaching quality.

#### 2.5.2. Caregiver Measures

##### Caregiver Characterization Measures

Family demographics. Caregivers completed a Child and Family Demographics Questionnaire at the study intake, including demographic information about the child and family and the child’s developmental history. This measure was developed by the authors for the purposes of this study.

##### Caregiver Dependent Variables

PI Parent Intervention Fidelity. PI fidelity definitions were used to evaluate parents’ use of PI components during parent–child interactions at entry and exit. Definitions were utilized from the Project ImPACT Intervention Fidelity Checklist (Ingersoll & Dvortscak, 2019). The PI fidelity measure includes 22 items that comprise five domains reflective of PI strategies (Focus on Your Child; Adjust Your Communication; and Create Opportunities, Teach New Skills, and Shape the Interaction). Items are rated on a 1–5 Likert-type scale (1 = minimal or no use of the strategy throughout the observation; 5 = correct use of the strategy at least 80% of the time throughout the entire observation). Interrater agreement within 1 point across items ranged from 81% to 100%, with a mean interrater agreement of 94%. Mean scores for each of the domains were calculated across the individual items and compared between services-as-usual and PI groups.

Self-Efficacy for Parenting Tasks Index–Toddler Scale. The Self-Efficacy for Parenting Tasks Index—Toddler Scale (SEPTI-TS; Coleman & Karraker, 2003) was used to examine parents’ feelings on managing day-to-day life with their young child. The SEPTI-TS is a parent-report questionnaire consisting of 26 items that measure parents’ confidence in managing common parenting tasks with toddlers across four domains: nurturance, discipline, play, and routine. Items are rated on a 6-point Likert scale ranging from 1 (very ineffective) to 6 (very effective), with higher scores indicating greater parenting self-efficacy. The SEPTI-TS has demonstrated good internal consistency and construct validity in prior research with parents of young children (Coleman & Karraker, 2003). The total scores (sum of items) for each domain were utilized at intake and exit for analyses.

Parenting Stress Index—Short Form. Parenting stress was assessed with the Parenting Stress Index–Short Form—4th Edition (PSI-SF; Abidin, 2012), a 36-item parent-report measure. PSI-SF contains three subscales (Parental Distress, Parent–Child Dysfunctional Interaction, and Difficult Child) and a Total Stress score. Parents rate items on a 5-point Likert scale ranging from 1 (strongly disagree) to 5 (strongly agree), with higher scores reflecting greater parenting stress. The PSI-SF has demonstrated strong internal consistency, test–retest reliability, and validity across diverse samples of parents of young children (Abidin, 2012; Reitman et al., 2002). The subscale scores and total scores at entry and exit were examined in the current analyses.

Parent Interactions with Children: Checklist of Observations Linked to Outcomes. The Parent Interactions with Children: Checklist of Observations Linked to Outcomes (PICCOLO; Roggman et al., 2013) was conducted at intake and exit. It is an observational measure in which a parent–child interaction is video-recorded, and trained observers code specific parenting behaviors known to predict children’s early social, cognitive, and language development. Specifically, the PICCOLO examines four domains of parenting behaviors, including affection, responsiveness, encouragement, and teaching. Each domain comprises seven to eight individual items that are rated on a 0–2 scale, where 0 = absent, 1 = rarely/briefly, and 2 = frequently. Higher scores are indicative of more developmentally supportive parent–child interactions. ICCs for individual items ranged from 0.68 to 0.81, with an average ICC of 0.79, and are considered good according to current standards (Cicchetti, 1994). The PICCOLO demonstrates strong reliability and both construct and predictive validity. Mean scores for each domain (affection, responsiveness, encouragement, and teaching) were calculated at entry and exit and utilized in analyses.

#### 2.5.3. Child Measures

##### Child Characterization Measures

Autism characteristics. Autism characteristics were assessed with the Telehealth Evaluation of Developmental Disorders—Autism Spectrum Disorder—Pediatrics (TELE-ASD-PEDS; Corona et al., 2020). The TELE-ASD-PEDS is a clinician-administered, telehealth-based assessment designed to evaluate autism-related behaviors in toddlers and young children. It includes structured prompts and caregiver–child interaction tasks that allow trained clinicians to rate core social communication and restricted/repetitive behaviors. Items are rated on a 0–3 scale, with higher scores indicating greater autism-related behaviors. The TELE-ASD-PEDS has demonstrated good interrater reliability and concurrent validity with gold-standard in-person assessments and feasibility for remote administration (Corona et al., 2024). In addition to the TELE-ASD PEDS, caregivers reported on their child’s behavior using the Toddler Autism Symptom Inventory (TASI; Coulter et al., 2021). The TASI is a parent-report questionnaire developed for early identification of autism spectrum disorder in toddlers. It assesses a broad range of social communication behaviors, restricted/repetitive behaviors, and other developmental features relevant to autism. Items are rated on a Likert scale, with higher scores reflecting greater endorsement of autism characteristics. For the purposes of the current study, information collected from both the TELE-ASD-PEDS and TASI was used to inform completion of a DSM-5 ASD Checklist for each child by the assessor. Given the available information, participants were considered to meet DSM criteria for autism if the assessor endorsed all three items in the Social Communication portion of the DSM-5 checklist (Criteria A; Social Communication) as well as at least two of the four items in Criteria B (Restricted/Repetitive Behaviors). This approach was necessary due to the COVID-19 pandemic and the impossibility of consistent in-person assessments for the purposes of research. Additionally, the sample in the current study is intended to be the population of children receiving early intervention services for social communication. An autism diagnosis was not necessary for inclusion in the study but was rather used to characterize the sample. Additionally, recent research has supported the use of these tools to examine characteristics of autism in young children (Corona et al., 2024; Gangi et al., 2025).

Developmental Profile—Cognitive Scale. Cognitive development was assessed using the Cognitive scale of the Developmental Profile, Third Edition (DP-3; Alpern, 2007). The DP-3 is a standardized caregiver-report instrument designed for children from birth through 12 years. The Cognitive scale includes items that assess reasoning, memory, problem-solving, and other age-appropriate intellectual abilities. The DP-3 Cognitive scale demonstrates strong reliability and validity in assessing early cognitive development (Alpern, 2007). Children’s standard scores were utilized in the current analyses.

##### Child Dependent Variables

Developmental Profile—Communication Scale. Communication abilities were assessed using the Communication scale of the Developmental Profile, Third Edition (DP-3; Alpern, 2007). The DP-3 is a standardized caregiver-report measure for children from birth through 12 years. The Communication scale includes items assessing both receptive and expressive language skills, such as vocabulary knowledge, comprehension, and verbal expression. The DP-3 Communication scale has demonstrated good reliability and validity for evaluating early communication development (Alpern, 2007). Children’s standard scores were utilized in the current analyses.

Communication and Symbolic Behavior Scales—Developmental Profile—Infant–Toddler Checklist. Parents reported on their child’s early social communication skills through the Communication and Symbolic Behavior Scales—Developmental Profile—Infant–Toddler Checklist (CSBS DP ITC; Wetherby & Prizant, 2002). The CSBS DP ITC is a 24-item caregiver-report screening tool designed for children 6 to 24 months of age, with applicability up to 24–36 months for children with developmental delays. The measure contains three domains: social communication, speech, and symbolic functioning. Items are rated based on frequency or presence/absence of specific behaviors. The CSBS DP ITC has demonstrated good reliability, validity, and sensitivity in identifying children at risk for communication and developmental delays (Wetherby & Prizant, 2002). For intake and exit, summed items in each domain and a total score were utilized in the current analyses.

Adaptive skills. The Vineland-3 (VABS-3) Comprehensive Parent Interview (VABS-3; Sparrow et al., 2016) was conducted at intake and exit. VABS-3 has been validated with children with developmental disabilities and is applicable to children from birth through 18 years, 11 months. Standardization included national samples of children with and without disabilities. The scales yield normative standard scores (M = 100; SD = 15) that indicate level of functioning and can be used for comparison across groups. Standard scores for the communication and socialization domains were utilized in analyses, in addition to the overall adaptive behavior composite standard score.

### 2.6. Coder Training

Research staff and interns were trained to score each of the behavioral coding schemes utilized in the study. For each coding scheme, gold-standard training videos were created through consensus scoring involving experienced clinicians on the research team. Coders met a reliability criterion of 80% or greater score agreement on three consecutive videos prior to beginning independent coding. Thereafter, approximately 30% of videos were double-coded in each scheme to examine reliability statistics on an ongoing basis. Coders were naïve to the study condition and time period of the videos.

### 2.7. Analyses

Multilevel models using full information maximum likelihood estimation were used to examine the target predictive models. Analyses were performed using MPlus, Version 8 and SPSS, Version 30 statistical software. Missing data were accounted for using the full information maximum likelihood approach implemented in MPlus.

A 3-level nested data structure (level 1 = time, level 2 = caregiver/child, level 3 = provider) was specified for the caregiver and child outcomes model. Time between assessments, whether the child met DSM criteria for autism, and child cognitive scores were included as covariates in the child outcome models.

Unfortunately, many of these analyses were underpowered, particularly for higher-order interaction terms. By definition, nested data structures have built-in dependencies in the data. This can be quantified by the intraclass correlation coefficient (ICC). The ICC was substantial at the program level for many of the target variables, thus reducing the effective sample size and reducing power. Moreover, there was a substantial difference in the size of the intervention and control groups. This further increases the standard error terms of statistical tests and reduces power. Finally, missing data was prevalent, particularly for variables assessed at follow-up. While a modern missing data approach (i.e., full information maximum likelihood) was used to estimate models, substantial missing data can result in unstable parameter estimates and inflated standard errors.

## 3. Results

### 3.1. Service Provider Results

Results indicated that the use of parent coaching practices in early intervention sessions differed between groups. Specifically, ratings for use of effective parent coaching strategies increased after providers had received training in PI, B = 1.30, *p* < 0.001. Providers received higher mean PEACE scores when delivering PI (M = 3.46; SE = 0.11) than when delivering services-as-usual (M = 2.17, SE = 0.08).

Service providers reported high levels of satisfaction with the PI training, strong beliefs in the impact of the model, and favorable ratings of implementation, with mean ratings on all subsections above 4.5/5. Providers most frequently endorsed following the child’s lead (78%) and using animation (66%) as the most useful strategies, and they most frequently highlighted playful obstruction (56%) and modeling and expanding communication (45%) as the most difficult to teach parents. Service providers unanimously stated that they would recommend the training and PI itself to others (though 15% of providers commented that they would do so outside the context of a research study, as they found the timelines long and/or required activities burdensome).

### 3.2. Caregiver Results

Table 1 provides demographic information for participating caregivers.

Table 2 includes caregiver fidelity scores across each domain. Caregivers demonstrated low to moderate levels of fidelity across the domains of PI, with most averages in the 2–3 range (out of 5). Strategies related to creating opportunities for children to respond and prompting and reinforcement received lower scores (at entry and exit) than other domains. There were no differences between groups in caregivers’ use of PI strategies from beginning to the end of services received (*p* > 0.05).

**Figure 3:**
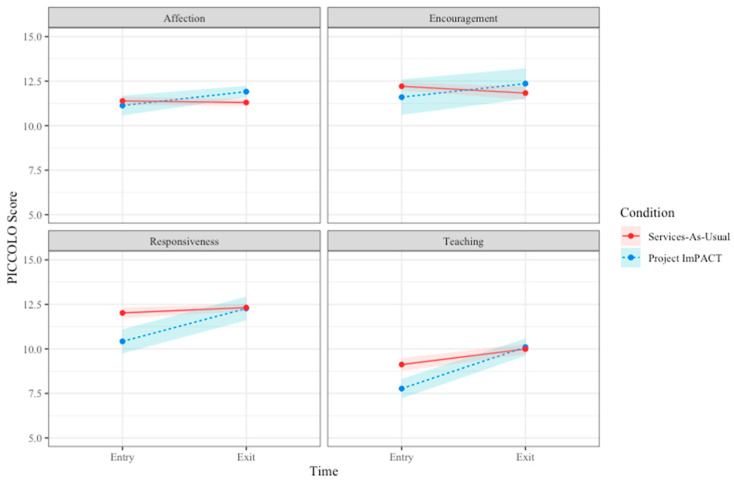
Caregiver PICCOLO Scores from Entry to Exit Across Groups.

As rated on the PICCOLO, parents who received PI showed a significant increase in Responsiveness and Teaching in their interactions with children, while parents in the services-as-usual group demonstrated no change (B = 1.56, *p* < 0.05 and B = 1.47, *p* < 0.05, respectively). PICCOLO scores in the Affection and Encouragement domains did not indicate change in either group (*p* > 0.05). PICCOLO results are represented in Figure 3 and Table 2.

Neither the result of the PSI nor caregivers’ self-efficacy demonstrated change in either group (*p* > 0.05). Table 2 includes caregiver scores for both these measures.

### 3.3. Child Results

Multilevel modeling analyses did not reveal any statistically significant condition effects between children who received PI and services-as-usual (*p* > 0.05). Children in both groups demonstrated significant growth over time in both measures of communication skills (VABS-3 Communication (B = 3.15, *p* < 0.05) and DP-3 Communication (B = 4.25, *p* > 0.001)), as well as CSBS DP ITC raw scores (B = 4.88, *p* > 0.001). Social skills demonstrated a decrease over time in both groups (VABS-3 Socialization (B = −0.37, *p* > 0.05)). Child DP-3 Communication Standard scores also increased from exit to follow-up (B = 4.4; *p* < 0.001) for both groups. No other child measures demonstrated significant change. Table 3 includes child scores for both groups across timepoints.

## 4. Discussion

The current community trial examined the effectiveness of PI when delivered by community-based EI providers to toddlers with social communication challenges. Overall, results indicate that community providers successfully improved their use of high-quality caregiver coaching strategies after PI training and that caregivers who received PI demonstrated improvements in key aspects of parent–child interaction quality. However, child-level outcomes did not differ significantly between the PI and SAU conditions, suggesting that while implementation at the provider and caregiver levels was feasible and associated with proximal benefits, differential developmental gains at the child level may require a higher number of participants to increase power and/or examination over a longer time scale to see measurable effects in community settings and/or more nuanced measurement.

Findings demonstrated significant improvements in provider use of evidence-based coaching strategies following PI training, with considerable differences in PEACE scores after service providers had received PI training. These results replicate and extend the findings from the earlier quasi-experimental pilot of PI for Toddlers (Stahmer et al., 2020), which showed similar improvements in provider coaching quality when PI was implemented in community EI contexts. The consistency of these findings provides support for the feasibility of training community EI providers to deliver evidence-based caregiver-mediated interventions. This is particularly noteworthy, as many service providers do not have prior experience with manualized NDBI approaches, and parent coaching is not often implemented in usual care contexts (Pellecchia et al., 2023). Additionally, a recent study of PI in EI services using detailed measures found a link between higher provider coaching fidelity and caregiver use of PI strategies related to creating opportunities and teaching new skills (Pickard et al., 2025). The results suggest that a structured, multi-component training model that includes both group workshops and individualized coaching may effectively build provider capacity for caregiver coaching, aligning with best-practice recommendations for EI (Dunst & Bruder, 2015; Friedman et al., 2012).

At the caregiver level, family participation in PI was associated with greater increases in responsive and teaching behaviors during parent–child interactions compared to caregivers in the SAU condition. These findings are consistent with prior literature demonstrating that parent-mediated interventions can enhance caregiver use of developmentally supportive strategies that, in turn, promote early communication development (Conrad et al., 2021; Reichow et al., 2024). The improvements in responsiveness and teaching are particularly notable given that these behaviors are central mechanisms through which NDBIs are hypothesized to influence child outcomes (Schreibman et al., 2015).

Despite these differences in overall developmental support, however, no differences were observed in caregiver fidelity ratings using the PI Fidelity Checklist. While this is consistent with our prior pilot study (Stahmer et al., 2020), it remains somewhat surprising that there are changes in the more distal parent responsiveness measure but not the more proximal fidelity tool. This may suggest that while caregivers improved their overall interactive quality (as reflected in PICCOLO scores), they may not have fully integrated specific PI strategies to criterion within the short intervention period. These findings may raise questions related to the way providers are teaching parents about the specific PI strategies or some aspects of the strategies that are most salient to parents. Ultimately, it is likely that the PICCOLO and PI Fidelity Checklist are measuring different constructs of parents’ behavior towards their children. While the PICCOLO is assessing more general domains of parent responsiveness, the PI Fidelity Checklist appears to examine technical application of individual techniques. Further research should explore the potential linkage and relationship between broad parent responsiveness versus the application of NDBI strategies specifically and the unique contributions each may have on children’s development. While caregivers in the current study are not showing change in specific strategies at a level that is detected by the fidelity tool, they are changing the way they interact with their child in ways that are likely to lead to more positive interactions, which has been identified as a potential mechanism of change supported by PI (Frost & Ingersoll, 2025).

There were no significant differences between groups in child social or communication outcomes. It remains encouraging that community-based EI is promoting developmental progress such that children in both conditions demonstrated growth across multiple measures after receiving services (e.g., DP-3 Communication, VABS-3, CSBS DP ITC). These findings are consistent with well-established research demonstrating that early intervention services can support communication development in toddlers with delays (Estes et al., 2015; Zablotsky et al., 2019). The decrease in social skills standard scores demonstrated in both groups is possibly a result of the increased social demands as children progress through toddlerhood, and more focus is needed on how to best support children in this domain. A recent single-group study of PI in EI systems also failed to find a relationship between caregiver fidelity to PI and social communication outcomes (Pickard et al., 2025).

It remains surprising that demonstrated changes in provider and parent behavior failed to translate into differential outcomes for the child in the present study. Larger, fully powered data sets and more fine-grained measurement of parent behavior may be needed to understand the specific parent behaviors that are associated with change in child-level outcomes. It is likely that service provider- and caregiver-level gains precede measurable child-level differences, such that the longer follow-up on children’s outcomes would demonstrate differences between groups. This is plausible given the expected benefit of parent-mediated intervention, as the increased amount of time that caregivers and children spend together increases the potential dosage of intervention received in the long term. The 4-month period in the current study may have been too brief to see the impacts of changes in caregiver behavior on children. Prior longitudinal research on caregiver-mediated interventions suggests that improvements in parent fidelity and responsiveness can mediate child developmental outcomes over longer time frames (Conrad et al., 2021; Reichow et al., 2024).

The substantial disruption to EI systems and families’ lives during the COVID-19 pandemic may have also contributed to the lack of findings, particularly in terms of missing data and difficulties with recruitment to engage a well-powered sample as originally planned. The shift to an all-virtual study protocol, particularly for measurements of change over time, may have diluted the effectiveness of the services offered and introduced bias into the assessment process through exclusive reliance on caregiver reports. It is strongly recommended that future research aim to replicate an examination of the effectiveness of NDBIs in community settings under more typical service conditions, including in-person, external assessment of child-level variables.

Implementation data and provider satisfaction ratings underscore that PI can be feasibly integrated into publicly funded community EI systems. Providers expressed high satisfaction with the training structure and perceived it as relevant to their day-to-day work with families. The hybrid training model of combining asynchronous learning with small-group, interactive practice and individualized coaching appears to support skill acquisition while accommodating the logistical demands of community service delivery. These findings align with implementation science frameworks emphasizing co-development, contextual fit, and ongoing support as critical components for sustaining evidence-based practices in real-world systems (Brookman-Frazee et al., 2012a; Pellecchia et al., 2023).

Several limitations must be considered. First, although the study used a randomized waitlist-control design, the nested structure and unequal group size reduced statistical power for detecting higher-order effects. This is particularly noteworthy at the child level, where the study failed to demonstrate differential outcomes across PI and SAU groups. Second, the COVID-19 pandemic required a rapid shift to virtual service delivery, altering the intervention context in ways that may have affected both implementation and outcomes. As discussed previously, because of COVID-19, the battery of measures utilized in the current study relied heavily on caregiver reports and remote observation, particularly for child outcomes. While original study protocols called for direct behavioral observation and more thorough characterization of children, changes were necessary due to the need for remote administration for much of the data collection period. This shift ultimately reduced the quality of assessment conducted for the study and required reliance on potentially biased caregiver reports, which may have contributed to the lack of effects identified.

Although the services examined were all delivered within community-based agencies and by providers who routinely work with families, participation in the research component was entirely voluntary. Only those agencies and providers who were willing and able to take on the additional responsibilities of completing study questionnaires, submitting videos, and maintaining communication with the research team are represented here. This opt-in process likely excluded individuals or organizations with fewer resources, heavier caseloads, or less interest in research participation and/or receiving training in PI. Moreover, even within participating providers, many families receiving services chose not to participate in the research study, often due to time constraints, competing demands, or limited interest in research involvement. This additional layer of self-selection further narrows the sample and underscores the possibility that the families who did take part may differ in important ways from those who declined. Overall, this structure may have resulted in a sample that is not fully representative of the broader EI service community at either the provider or family level. Future explorations of NDBIs in community settings should aim to reduce the burden of participating in the research beyond the typical receipt of EI services in order to address this limitation.

Future studies should replicate and extend these findings under typical service conditions and examine the sustainability of provider coaching skills over time. Future longitudinal analyses should explore whether early changes in caregiver interaction quality predict later child communication and social outcomes, thereby clarifying mechanisms of change in community-based NDBI implementation. Additional work is also needed to determine optimal training dosage and supervision models for large-scale community dissemination. Though service providers generally recommended the PI training, several commented on the length of time it took to complete, and anecdotally, agency leaders continually mention this challenge in the community. Future research should inform what training models for NDBIs can balance high-quality instruction that will support provider behavior change with feasibility and acceptability for community agencies.

This study contributes important evidence supporting the scalability of caregiver-mediated NDBIs in public early intervention systems. PI training led to substantial improvements in provider use of evidence-based caregiver coaching strategies and enhanced caregiver–child interaction quality, demonstrating that community-based providers can effectively implement this model with fidelity and thereby positively influence caregiver behavior with their toddler.

## Figures and Tables

**Table 1:** Caregiver and child demographic information.

	Project ImPACT (n = 78)	Services-As-Usual (n = 47)	Total (n = 125)
	M (SD)	M (SD)	M (SD)
Child Age (months)	25.20 (5.3)	25.53 (4.4)	25.41 (4.7)
Child Sex Male	61%	55%	58%
% Met DSM Criteria	52%	49%	50%
DP-3 Cognitive Score	75.06 (21.01)	84.4 (24.41)	80.86 (23.57)
CSBS Total Score	35.97 (1.67)	38.45 (1.41)	37.83 (12.09)
Vineland ABC SS	72.15 (2.53)	70.44 (1.78)	88.96 (13.25)
Communication SS	66.13 (3.34)	66.19 (2.97)	84.00 (12.42)
Social SS	75.88 (2.52)	74.11 (1.97)	91.35 (12.63)
	%	%	%
Parent Race/Ethnicity (May Select more than 1)			
Hispanic/Latine	49	38	42
American Indian or Alaska Native	6	1	3
Asian	12	5	8
Black or African American	6	4	5
Native Hawaiian or Pacific Islander	0	3	2
White	51	59	56
Other	4	8	6
Maternal Education			
Did Not Complete High School	6	5	6
High School/GED	33	20	25
Associate’s or Vocational Degree	18	22	21
Bachelor’s Degree	18	22	21
Master’s Degree	4	13	10
Doctoral Degree	6	5	6
Preferred Language			
Spanish	24	24	24
English	76	76	76
Annual Family Income			
Below $49,999	22	21	21
$50,000–$89,999	20	13	16
Above $90,000	24	36	31
Prefer Not to Answer	33	32	32

**Table 2:** Caregiver Measures Across Timepoints.

Parent Outcomes	PI		SAU	
Measure/Subscale	Intake M (SD)	Exit M (SD)	Intake M (SD)	Exit M (SD)
Fidelity				
Focus	3.37 (0.50)	3.60 (0.60)	3.32 (0.56)	4.27 (0.52)
Adjust	2.97 (0.54)	3.20 (0.46)	2.76 (0.58)	3.55 (0.66)
Create	2.23 (0.94)	2.62 (1.06)	2.21 (0.96)	2.94 (1.07)
Teach	3.29 (0.39)	3.54 (0.42)	3.26 (0.50)	3.39 (0.42)
Shape	2.13 (0.78)	2.62 (1.02)	2.13 (0.86)	2.37 (0.99)
Overall	3.00 (0.44)	3.28 (0.47)	2.94 (0.44)	3.12 (0.46)
PICCOLO				
Affection	11.13 (0.56)	11.91 (0.33)	11.39 (0.23)	11.30 (0.20)
Responsiveness	10.42 (0.68)	12.27 (0.67)	12.02 (0.30)	12.32 (0.20)
Encouragement	11.60 (1.00)	12.36 (0.87)	12.21 (0.24)	11.83 (0.38)
Teaching	7.77 (0.55)	10.10 (0.49)	9.12 (0.36)	9.99 (0.30)
Parent Stress Index				
Parental Distress	46.56 (1.24)	45.85 (1.44)	47.49 (0.91)	47.00 (0.76)
Parent–Child Dysfunction	47.24 (0.81)	46.59 (1.20)	45.88 (0.91)	45.85 (0.83)
Difficult Child	43.23 (1.26)	42.60 (1.49)	42.26 (1.03)	42.07 (0.90)
Total Stress	136.20 (2.86)	134.47 (3.62)	135.68 (2.44)	134.80 (2.14)
Self-Efficacy in Parenting				
Overall	128.99 (1.84)	130.19 (1.77)	128.06 (1.42)	126.88 (1.46)

**Table 3:** Child Measures Across Outcomes.

	PI		SAU	
Measure/Subscale	Intake M (SD)	Exit M (SD)	Intake M (SD)	Exit M (SD)
**Vineland**				
Communication SS ***	66.13 (3.34)	67.02 (3.97)	66.19 (2.97)	69.99 (3.31)
Socialization SS	75.88 (2.52)	73.10 (2.41)	75.11 (1.97)	73.68 (2.32)
ABC Composite SS	72.15 (2.53)	72.51 (2.51)	70.44 (1.78)	72.80 (2.45)
CSBS—DP—ITC *				
Social	14.98 (5.52)	14.31 (3.64)	15.93 (6.14)	16.25 (5.39)
Speech	5.91 (3.87)	7.02 (3.66)	7.42 (2.90)	8.98 (3.25)
Symbolic	10.80 (2.86)	11.41 (1.94)	11.44 (5.12)	12.69 (4.10)
Total	35.97 (1.67)	42.29 (2.06)	38.45 (1.41)	42.95 (1.44)
DP-3 **				
Communication SS	74.62 (3.69)	80.91 (3.63)	77.76 (1.91)	81.80 (2.42)

* CSBS = Communication Symbolic Behavior Scales—Developmental Profile—Infant–Toddler Checklist; ** DP-3 = Developmental Profile 3; *** SS = Standard Score.

## Data Availability

The raw data supporting the conclusions of this article will be made available by the authors on request.

## Abbreviations

Table following abbreviations are used in this manuscript:

NDBI	Naturalistic developmental behavioral intervention
EI	Early intervention
PI	Project ImPACT (Improving Parents as Communication Teachers)
BRIDGE	Bond, Regulate, Interact, Develop, Guide, Engage
SAU	Services-as-usual
DP-3	Developmental Profile-3
PEACE	Parent empowerment and coaching in early intervention
ICC	Intraclass correlation coefficients
SEPTI-TS	Self-efficacy for parenting tasks index: Toddler scale
SS	Standard Score
PSI-SF	Parenting Stress Index—Short Form
PICCOLO	Parenting Interactions with Children: Checklist of Observations Linked to Outcomes
TELE-ASD-PEDS	Telehealth Evaluation of Developmental Disorders—Autism Spectrum Disorder
TASI	Toddler Autism Symptom Inventory
CSBS DP ITC	Communication and Symbolic Behavior Scales—Developmental Profile—Infant–Toddler Checklist
VABS-3	Vineland Adaptive Behavior Scales-3
